# Army Medical Board

**Published:** 1850-04

**Authors:** 


ARMY MEDICAL BOARD.

A Board of Army Surgeons, for the examination of Assistant Surgeons for promotion, and of applicants for appointment to the Medical Staff of the Army, is to convene in New York city on the 15th of May next, and will probably continue in session during three or four weeks. The National Intelligencer says:—

Applications must be addressed to the Secretary of War; must state the age and residence of the applicant; and must be accompanied by respectable testimonials (mere references are not sufficient) of his possessing the moral and physical qualifications for filling creditably the responsible station, and for performing ably the arduous and active duties of an officer of the Medical Staff.

